# Progress in Surgery

**Published:** 1896-08-08

**Authors:** 


### PROGRESS IN SURGERY.

### RECTAL SURGERY.

**Fistula in Ano.**—The possibility of obtaining primary union after excision of the fistulous tracks, and the damage done to the sphincter in operating for fistula, were discussed at the Medical Society of Bellevue Hospital[1]. Dr. Blair Gibbs pointed out the great damage sometimes inflicted on the sphincter by the multiple incisions needful in laying open all the lengthy and tortuous fistulous tracks and the consequent distressing incontinence, but he failed to suggest any method of avoiding such a result if all the fistulous tracks were laid open, as is the usual practice. His experience of trying to excise the fistulous tracks and then obtain primary union after suturing the wounds, was not a satisfactory one. Like many other surgeons he has found the rectum a potent source of infection for the wounds, which had failed to unite. No doubt if these long multiple fistulæ could be excised, and primary union of the wound obtained, the integrity of the sphincter might be saved in cases where now incontinence results. Other speakers agreed with Dr. Gibbs as to the great difficulty of obtaining primary union after excision of the fistula, and Dr. Johnson considered that it was often impossible to follow up or find all the fistulous channels. Dr. Porter, on the other hand, had been very successful, obtaining primary union after excision of the fistulous tracks. He thoroughly washed out the rectum first, and packed it with iodiform gauze after the operation. Dr. Robert Morris only attempted excision and suture in non-tuberculous cases when the surrounding infiltration was not great. In such he had often been successful. He injected plaster of Paris along the tracks to discover all their ramifications. After suturing the wounds he covered them with aristol or finely-powdered iodoform, so as to form a protecting barrier against microbic infection.

**Carcinoma of the Rectum.**—A method of excising cancer of the rectum has been devised by Quénu[2], in which there can be no escape of fæces when the bowel is divided high up in the sacral operation for removal of cancer situated in the upper part of the rectum. First iliac colotomy is performed. Then partly by perineal incision, and partly by removal of a portion of the sacrum, the rectum is isolated, and two ligatures passed round the bowel above the disease, dipping into the coats, but not into the lumen, and the bowel is then divided between them. The upper end, with the ligature still surrounding it, is brought out and fixed in the upper angle of the wound, and the ligature is not removed, but allowed to cut its way out, which it usually does in five or six days, when the surrounding tissues have to a large extent united.

Heidenham, who has done so much for the surgery of cancer of the breast, contributes a paper on Kraske's operation for rectal carcinoma.[3] He considers that the only conditions which contra-indicate excision are intimate adhesions to the prostrate, bladder, or ureters, or extensive invasion of the ischio-rectal fossa. He thinks it possible to differentiate the diseased from the sound tissues, and to control the hæmorrhage, much better in the sacral than in the perineal operation. Whenever, on account of extreme narrowing of the bowel, it cannot be thoroughly emptied before operation he recommends a preliminary colotomy. The sacral method allows of thorough removal of lymphatic structures, in the hollow of the sacrum, in the same way that they are from the axilla in cancer of the breast. If the portion of bowel surrounded by the sphincter is sound it can be preserved, and the bowel above the excised portion brought down and sutured to it if possible. Good results have been obtained by Witzel by bringing the upper end of the divided bowel through a slit in the gluteus muscle and suturing it to the skin, so as to form a new sphincter in cases where the old one has had to be removed. If this is not done, and the intestine cannot be drawn down far enough to attach it to the skin when the anus has been removed, it must be brought out at the upper end of the sacral wound. In cases where the patient is much shocked the operation may be performed in two stages, first the rectum isolated and surrounded by gauze, and then later on the portion affected excised.

Mr. Watson Cheyne, in his Lettsonian lectures on "The Objects and Limits of Operations for Cancer,"[4] discusses the relative advantages of colotomy and excision of cancer of the rectum. He agrees with the English school of surgery as opposed to the German, and would exclude from the radical operation cases of rapid growth, cases where the disease forms large masses in the intestine or is deeply ulcerated, cases where it has passed through the walls of the rectum and invaded surrounding parts as indicated by fixidity, and rapidly-growing tumours high up, even though not yet fixed. He considers that in none of these is there any real prospect of cure or of marked prolongation of life. On the other hand, in colotomy we have, he believes, a means of relieving the patient's sufferings, and of giving him as long a period of life with practically no risk. He has found the mortality of perineal excision of cancer of the rectum, taken from a large number of reports, to be about 8 per cent., but after the sacral operation 18 to 20 per cent. The mortality of inguinal colotomy is only 2 or 3 per cent. when done before obstruction comes on. Out of 100 cases of excision of the growth, in which a considerable time had elapsed since the operation, 61 were no better off than if they had been left alone, and 27 out of the 61 died. Thirty-three gained advantage from the operation, and most of them very marked advantage, much greater than colotomy could have given. There are, however, some facts which would encourage excision of rectal cancer. In Czerny's statistics ten were well after three years. Metastases are late in occurring, for out of 47 long-standing cases of rectal cancer nearly half were found free from metastatic growths at the post-mortem examination. Mr. Allingham also discusses the desirability of performing excision or colotomy in this disease.[5] He considers that the older the patient becomes the less rapidly does the malignant disease grow, and the less likely is it to recur. If the age of his patient does not exceed forty-five years, the disease will at once return after excision, and colotomy will be of very slight advantage; but for patients above sixty years of age excision, so long as we can get well above the growth, is a very satisfactory operation. Mr. Allingham does not consider Kraske's operation in as unfavourable a light as Mr. Watson Cheyne, for he says when the growth is some way up the bowel, but can be easily felt, and is freely movable, though its upper limit may not be readily definable, Kraske's operation may be performed if the patient consents to undergo it on learning of its serious nature and the danger he may incur. He does not consider that colotomy should ever be performed to divert the fæces, and thus try and stay the rapidity of the growth, for he does not believe such an end would be obtained. He thinks the operation should be reserved for cases of obstruction, or those attended with considerable suffering.

Quénu does not think[6] that the height reached by the growth, but the degree of mobility of the cancerous rectum on the neighbouring parts, constitutes the main point for consideration in deciding on the advisability of operating in rectal cancer; the only inoperable cases, he considers, are those in which the rectum is immovable.

A method of excising a cancerous rectum, described as abdomino-perineal, has been devised by Dr. Gaudier.[7] He first opens the abdomen and divides the bowel after double ligature, and then the upper part of the rectum is separated from surrounding parts. The lower part is then directed off the structures with which it is in contact by a perineal incision, and the whole rectum drawn out through the opening in the peritoneum. The operation has only once been performed on a patient; he died on the fifth day from disease in the chest. It is only intended for cases of cancer extending very high up the bowel.

**Hæmorrhoids.**—The following method for rendering the rectum as aseptic as possible has been recommended by Thénenard.[8] Milk diet for six days before operation, with purgation every other day, but not on the day before operation. On the two days preceding the operation a copious enema of boracic acid should be given, but not on the morning of the operation. Three 8-grain doses of B. naphthol, with an equal quantity of salicylate of bismuth, should be given daily. Both the skin around the anus (after shaving) and the interior of the lower end of the bowel (after dilatation of the sphincter) should be well cleansed. Iodoform gauze is loosely packed into the rectum after the operation. Quénu[9] operation for piles differs from Whitehead's, in that the mucous membrane of the lower end of the rectum is not removed but only dissected up from the subjacent tissue, and all the dilated veins found on its cut surface removed and also those in the submucous tissue from which it has been dissected. The mucous membrane is then replaced and stitched to the skin. A plug of gauze is inserted into the rectum in ordered to press the divided surfaces together.

Andrews[10] as the result of extensive correspondence with European and American surgeons has collected 201 cases in which disaster followed Whitehead's operation, the most frequent being incontinence of flatus and fæces. He considers that it is most important to preserve the mucous membrane just above the sphincter, as on its integrity depends the ability of the nervous mechanism to control the contraction of the sphincter when fæcal matter presses on this region. If it is removed all the "tactile" mechanism is destroyed, for it is contained in eight small papillæ surrounding the rectum just above the anus and in the columns of Morgagni, between the rectal pouches. He found that in many cases of Whitehead's operation there has been failure to secure union by first intention, and a circular ulcer has formed, ultimately resulting in stricture. He is strongly in favour of the clamp and cautery operation in preference to Whitehead's operation.

**Stricture of the Rectum.**—Mr. Christopher Heath[11] records a case of syphilitic stricture of the rectum, or at any rate non-malignant stricture, in which the bowel wall was perforated by a bougie. The stricture had been previously dilated by the finger, and the bougie softened by heat was passed without any force, or particular complaint on the part of the patient. It was left in for an hour, and a few hours after its withdrawal urgent symptoms set in and she died of peritonitis.

**Villous Tumour of Rectum.**—Mr. Paul, of Liverpool, has recently reported a case of this nature.[12] The patient was a woman aged fifty-six, who suffered from a feeling of bearing down, and a constant desire to defæcate with the passage of blood and mucous. The symptoms were of five years' duration. The growth formed a band two or three inches deep around the rectum. It was a typical villous papilloma. Recovery was satisfactory.

[1] N.Y. Med. Record, March 21, 1896. [2] Epitome B.M. Journal, Feb. 15, 1896, and Presse Méd., Nov. 9, 1895. [3] Epitome B.M. Journal, March 14, 1896; Fortschritte d. Med. Nov. 2, 1896. [4] B.M. Journal, March 7, 1896. [5] Lancet, April 25, 1896. [6] Medical Week, April 3. 1896. [7] Ibid. [8] Practitioner, March, 1896, p. 298. [9] Ibid. [10] Therapeutic Gazette, Dec. 16, 1895. [11] Clinical Journal, Jan. 22, 1896, [12] Med. Record, Feb. 15, 1896.

